# FOOD INSECURITY AND COGNITIVE TRAJECTORIES IN COMMUNITY-DWELLING OLDER ADULTS

**DOI:** 10.1093/geroni/igac059.528

**Published:** 2022-12-20

**Authors:** Boeun Kim, Sarah Szanton, Roland J Thorpe, Jr., Deidra Crews, Laura Samuel

**Affiliations:** Johns Hopkins University, Baltimore, Maryland, United States; Johns Hopkins University School of Nursing, Baltimore, Maryland, United States; Johns Hopkins University, Baltimore, Maryland, United States; Johns Hopkins University, Baltimore, Maryland, United States; Johns Hopkins University, Baltimore, Maryland, United States

## Abstract

Food insecurity, defined as limited access to nutritionally adequate and safe foods due to social and economic conditions, has adverse effects on physical health and well-being. However, it remains unclear whether food insecurity accelerates cognitive decline in older adults. This study examined the association of food insecurity with cognitive decline. We analyzed data from 4,508 community-dwelling participants in the National Health and Aging Trends Study (NHATS) collected from 2011 to 2020, a prospective cohort study of a nationally representative sample of Medicare beneficiaries ages 65 years and older. Food insecurity was measured using 5 items within functional, social support, and financial limitations domains. Immediate and delayed recall were assessed by a 10-item word-list memory task (range: 0—10). Executive function was evaluated by the Clock Drawing Test (range: 0—5). Each year’s cognitive functions were linked to the prior year’s food insecurity values. Weighted linear mixed effect models were fitted. Prevalence of food insecurity at baseline was 3.5% (95% CI: 2.9 — 4.3). Persons with food insecurity were more likely to have Black race or Hispanic ethnicity, low income, and less than high school education. Food insecurity was associated with faster decline in executive function accounting for demographic and socioeconomic characteristics: the average difference, over 1-year, in executive function score between people exposed to and not exposed to food insecurity was 0.038 points (95% CI: -0.072 — -0.003). Food assistance programs or increasing healthy food access may reduce food insecurity and delay cognitive aging in community-dwelling older adults.

